# Infrared chemical imaging through non-degenerate two-photon absorption in silicon-based cameras

**DOI:** 10.1038/s41377-020-00369-6

**Published:** 2020-07-20

**Authors:** David Knez, Adam M. Hanninen, Richard C. Prince, Eric O. Potma, Dmitry A. Fishman

**Affiliations:** 1 Department of Chemistry, University of California, Irvine, CA 92697 USA; 2 Department of Biomedical Engineering, University of California, Irvine, CA 92697 USA

**Keywords:** Imaging and sensing, Infrared spectroscopy, Nonlinear optics, Mid-infrared photonics

## Abstract

Chemical imaging based on mid-infrared (MIR) spectroscopic contrast is an important technique with a myriad of applications, including biomedical imaging and environmental monitoring. Current MIR cameras, however, lack performance and are much less affordable than mature Si-based devices, which operate in the visible and near-infrared regions. Here, we demonstrate fast MIR chemical imaging through non-degenerate two-photon absorption (NTA) in a standard Si-based charge-coupled device (CCD). We show that wide-field MIR images can be obtained at 100 ms exposure times using picosecond pulse energies of only a few femtojoules per pixel through NTA directly on the CCD chip. Because this on-chip approach does not rely on phase matching, it is alignment-free and does not necessitate complex postprocessing of the images. We emphasize the utility of this technique through chemically selective MIR imaging of polymers and biological samples, including MIR videos of moving targets, physical processes and live nematodes.

## Mid-infrared imaging: silicon success

Standard silicon CCD cameras can be used for capturing mid-infrared chemical images by harnessing two-photon absorption within the camera’s image sensor. David Knez and coworkers from the University of California at Irvine, USA, used a mid-IR beam in combination with a near-infrared pump beam to induce a two-photon absorption process in silicon. The pump beam is sent straight to the camera, whereas the mid-IR beam is sent through the sample to be imaged prior to reaching the camera. Two-photon absorption of the pump and mid-IR light in the camera’s CCD sensor produces an image. The team successfully generated both static images and movies of various samples including polymer films, oil drops, a bee wing and live nematodes at several different mid-IR wavelengths.

## Introduction

Many fundamental molecular vibrations have energies in the mid-infrared (MIR) window—a wavelength region that stretches from approximately 2 to 10 μm. For this reason, the MIR range is of particular interest for spectroscopic imaging. The ability to generate images with chemical selectivity is of direct relevance to a myriad of fields, including the implementation of MIR-based imaging for biomedical mapping of tissues^1–3^, inspection of industrial ceramics^4^, stand-off detection of materials^5^, mineral sensing^6,7^, and environmental monitoring^8^, among others.

Given its unique analytical capabilities, it is perhaps surprising that MIR-based imaging is not a more widely adopted technology for chemical mapping. The relatively scarce implementation of MIR imaging has been due in part to the lack of bright and affordable light sources in this range, although recent developments in MIR light source technology have largely overcome this problem^9–11^. Nonetheless, a remaining limitation is the performance and high cost of the MIR cameras. Current cameras are based on low bandgap materials, such as HgCdTe (MCT) or InSb, which inherently suffer from thermally excited electronic noise^12^. Cryogenic cooling helps to suppress this noise, but it renders the MIR camera a much less practical and affordable detector than mature Si-based detectors for the visible and near-IR. Electronically cooled MCT detectors are a promising alternative, although the matrixes of such detector arrays are still of low density and are not yet on par with high definition Si-based CCD cameras.

Recognizing the attractive features of Si-based cameras, several strategies have been developed that aim to convert information from the MIR range into the visible/NIR range, thus making it possible to indirectly capture MIR signatures with a Si detector. A very recent development is the use of an entangled MIR/visible photon pair, which allows MIR imaging and microscopy utilizing nonlinear interferometry for detecting visible photons entangled to their MIR counterparts on a Si-based camera^13–15^. Another strategy accomplishes the MIR-to-visible conversion by using a nonlinear optical (NLO) response of the sample, such as in third-order sum-frequency generation (TSFG) microscopy^16^. Photothermal imaging, which probes the MIR-induced changes in the sample with a secondary visible beam, is another example of this approach^17–21^. An alternative but related method is the acoustic detection of the MIR photothermal effect, which has recently been demonstrated^22^. Another technique uses a nonlinear optical crystal placed after the sample to up-convert the MIR radiation with an additional pump beam through the process of sum-frequency generation (SFG)^23–29^. The visible/NIR radiation produced can be efficiently registered with a high bandgap semiconductor detector. Elegant video-rate MIR up-conversion imaging has recently been accomplished with a Si-based camera at room temperature, offering an attractive alternative to imaging with MCT focal plane arrays^30^. A possible downside of SFG up-conversion techniques is the requirement of phase matching of the MIR radiation with the pump beam in the NLO medium. This requirement implies crystal rotation to enable the multiple projections needed for capturing a single image and postprocessing for each measured frame for image reconstruction.

An alternative to utilizing an optical nonlinearity of the sample or a dedicated conversion crystal for indirect MIR detection (SFG up-conversion) is the use of the NLO properties of the detector itself. In particular, the process of non-degenerate two-photon absorption (NTA) in wide bandgap semiconductor materials has been shown to permit the detection of MIR radiation at room temperature with the help of an additional visible or NIR probe beam^31–34^. In NTA, the signal scales linearly with the MIR intensity with detection sensitivities that rival those of cooled MCT detectors^31^. Compared with SFG-based up-conversion, NTA does not depend on phase matching and avoids the need for an NLO crystal altogether, offering a much simpler detection strategy. Moreover, the nonlinear absorption coefficient drastically increases with the energy ratio of the interacting photons^35–39^, allowing detection over multiple spectral octaves. Although NTA has been shown to enable efficient MIR detection with single pixel detectors, its advantages have not yet been translated to imaging with efficient Si-based cameras. Here, we report rapid, chemically selective MIR imaging using NTA in a standard CCD camera at room temperature.

The nature of nonlinear absorption enhancement for direct-band semiconductors has been modelled with allowed-forbidden transitions between two parabolic bands^37–40^. The nonlinear absorption coefficient *α*_2_ for photon energies *ħω*_pump_ and *ħω*_MIR_ can be written as^40^:

1 $$\begin{array}{l}\alpha _2\left( {\omega _{\mathrm{p}},\omega _{{\mathrm{MIR}}}} \right) = K\frac{{\sqrt {E_{\mathrm{p}}} }}{{n_{\mathrm{p}}n_{{\mathrm{MIR}}}E_{\mathrm{g}}^3}}F\left( {x_{\mathrm{p}},x_{{\mathrm{MIR}}}} \right)\\ F = \frac{{\left( {x_{\mathrm{p}} + x_{{\mathrm{MIR}}} - 1} \right)^{3/2}}}{{2^7x_{\mathrm{p}}\left( {x_{{\mathrm{MIR}}}} \right)^2}}\left( {\frac{1}{{x_{\mathrm{p}}}} + \frac{1}{{x_{{\mathrm{MIR}}}}}} \right)^2,x_{\mathrm{p}} = \frac{{\hbar \omega _{\mathrm{p}}}}{{E_{\mathrm{g}}}},x_{{\mathrm{MIR}}} = \frac{{\hbar \omega _{{\mathrm{MIR}}}}}{{E_{\mathrm{g}}}}\end{array}$$

where *E*_p_ is the Kane energy parameter, *n*_p_ and *n*_MIR_ are refractive indices and *K* is a material independent constant. The function *F* accounts for the change in the nonlinear absorption as the ratio between the pump and MIR photon energies is adjusted, with dramatic enhancements when the pump energy is tuned closer to the bandgap energy *E*_g_. For an indirect bandgap semiconductor, such as Si, optical transitions can be understood as a nonlinear process that involves three interacting particles—two photons and a phonon. Several models have been considered to describe multiphoton absorption in Si, including earlier “forbidden-forbidden” models^41^ and more recently suggested “allowed-forbidden” and “allowed-allowed” pathways^42^. The latter two models agree well with degenerate absorption experiments^43^. For the case of NTA, experiments demonstrate enhancement behaviour similar to those seen in direct-bandgap semiconductors^44,45^, with the “allowed-allowed” pathways providing the best description^46^. Modest numbers of acquired and derived nonlinear absorption coefficients of only a few cm/GW have classified Si as a rather inefficient material for NTA. For this reason, attempts to develop MIR detection strategies based on Si detectors have been scarce^46^. In this work, we show that despite previous concerns, detecting MIR radiation through NTA in silicon is not only feasible but readily provides a very practical approach for MIR imaging with standard cameras.

## Results

### MIR detection with a Si photodiode

**Fig. 1:**
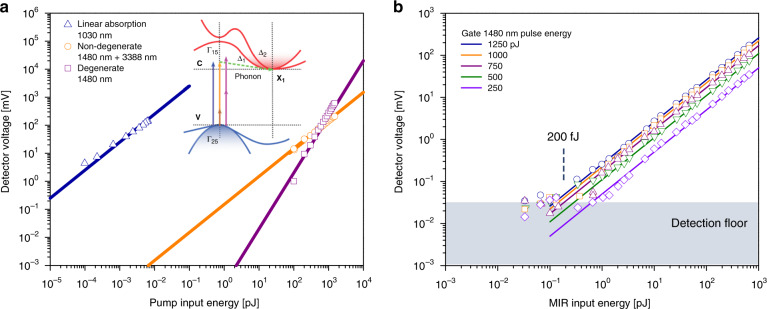
Detection of weak infrared radiation via non-degenerate two-photon absorption in a Si photodiode. **a** Linear absorption (blue) as a function of the pulse energy at 1030 nm, non-degenerate two-photon absorption (orange) as a function of the pump pulse energy at 1480 nm and degenerate two-photon absorption (purple) as a function of the pump pulse energy at 1480 nm. For the non-degenerate curve, the MIR pulse energy at 3388 nm was set at 0.65 nJ. Inset: proposed scheme of photon absorption in Si. **b** Full dynamic range for MIR detection with a detection floor of a 200 fJ picosecond pulse energy for the given detector parameters. Note that 1 V on the *y*-axis corresponds to 8.2 × 10^4^ electrons/pulse

We first discuss the utility of Si as a MIR NTA detector using picosecond pulses of low peak intensities. In Fig. 1a, we compare the linear absorption of 9708 cm^−1^ (1030 nm) photons by a standard Si photodiode with that of NTA for a 2952 cm^−1^ (3388 nm) MIR and 6756 cm^−1^ (1480 nm) pump pulse pair. Since the 1030 nm photon energy exceeds the Si bandgap energy (*E*_g_ ~1.1 eV (1100 nm)), strong one-photon absorption can be expected. Based on this measurement, the estimated responsivity is *R* = 0.2 A/W, close to the reported response for Si detectors at 1030 nm. In the NTA experiment, the MIR and pump photon energies add up to the same energy (9708 cm^−1^) as in the one-photon experiment, and thus, we may expect a response in Si, albeit weaker. The current photon energy ratio is *ω*_pump_*/ω*_MIR_ = 2.2. The NTA response is shown in orange and is compared with the degenerate two-photon absorption of the pump pulse. As expected, the NTA signal scales linearly with the NIR pulse energy. Previously reported values of *a*_*2d*_ ~ 2 cm/GW^43^ for the degenerate cases and *a*_*2n*_ ~ 5 cm/GW^46^ for the non-degenerate cases with comparable photon ratios agree well with our observations. Note that there is a regime where the NTA is stronger than the degenerate two-photon absorption of the pump using a 0.65 nJ MIR pulse at 3388 nm.

We next studied the sensitivity of MIR detection through NTA in Si. In Fig. 1b, the detected NTA signal is plotted as a function of the MIR pulse energy MIR (at 2952 cm^−1^) for various energies of the pump pulse. For these experiments, especially at higher NIR peak intensities, the degenerate contribution of the pump pulse has been subtracted using the modulation of the MIR beam and lock-in detection. We observe that the signal scales linearly with the MIR pulse energy for all settings. The minimally detectable MIR picosecond pulse energy is ~200 fJ using rather modest NIR pump peak intensities. In previous work with a direct large-bandgap GaN detector, a detection limit of 100 pJ was reported using femtosecond pulses and a photon energy ratio >10^31^. Here, we observe higher detection sensitivities in Si while using picosecond pulses and a much lower photon energy ratio. Such high detection sensitivities are remarkable and are due in part to the favourable pulse repetition rate (76 MHz) used in the current experiment, offering much better sampling than the kHz pulse repetition rates used previously. The strategy used here offers superior sensitivity, detecting 4 orders of magnitude smaller MIR peak intensities of 20 W/cm^2^ (with 0.09 MW/cm^2^ at 1480 nm pump pulse) versus 0.2 MW/cm^2^ (with 1.9 GW/cm^2^ at 390 nm pump pulse), as previously reported^31^. Given that the enhancement scales with the photon energy ratio, we may expect even greater sensitivities for experiments with higher pump photon energies and lower MIR photon energies, with a projected detection floor as low as a few tens of femtojoules (1 W/cm^2^).

### MIR spectroscopy with a single pixel Si detector

**Fig. 2:**
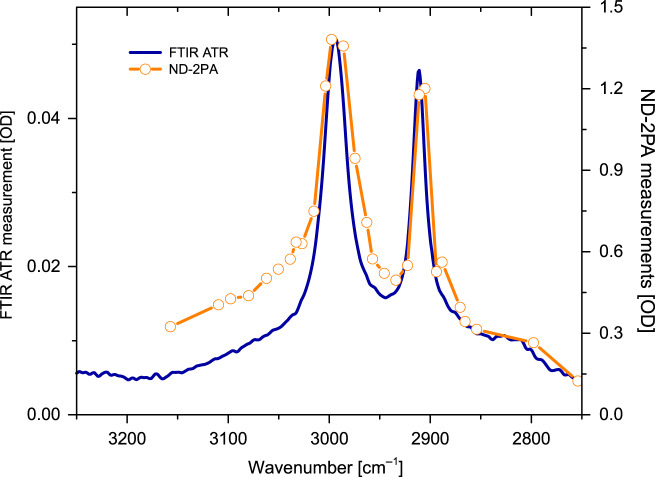
Absorption spectrum of dimethyl sulfoxide (DMSO) using non-degenerate two-photon detection for measuring the transmitted MIR radiation. The results are in excellent agreement with the spectrum obtained with conventional ATR-FTIR of bulk DMSO

As an example of the utility of MIR detection with a Si photodetector, we perform an MIR absorption spectroscopy experiment on a dimethyl sulfoxide (DMSO) film of a few tens of microns. For this purpose, we spectrally scan the MIR energy in the 2750–3150 cm^−1^ range and detect the MIR transmission via NTA on a Si photodiode. The spectral resolution is determined by the spectral width of the picosecond pulse (~5 cm^−1^). For these experiments, the MIR pulse was kept at 15 mW (~10 kW/cm^2^ peak intensity), while the NIR pump beam was set to 100 mW (66 kW/cm^2^). Because the pump and MIR pulses are parametrically generated by the same source, there is no temporal walk-off on the picosecond timescale while performing the scan. The resulting DMSO absorption spectrum shows the characteristic lines associated with the symmetric and asymmetric C–H stretching modes^47^, which corroborates the Fourier transform IR (FTIR) absorption spectrum (Fig. 2, see the section “Materials and methods”).

### MIR imaging through on-chip NTA in a CCD camera

**Fig. 3:**
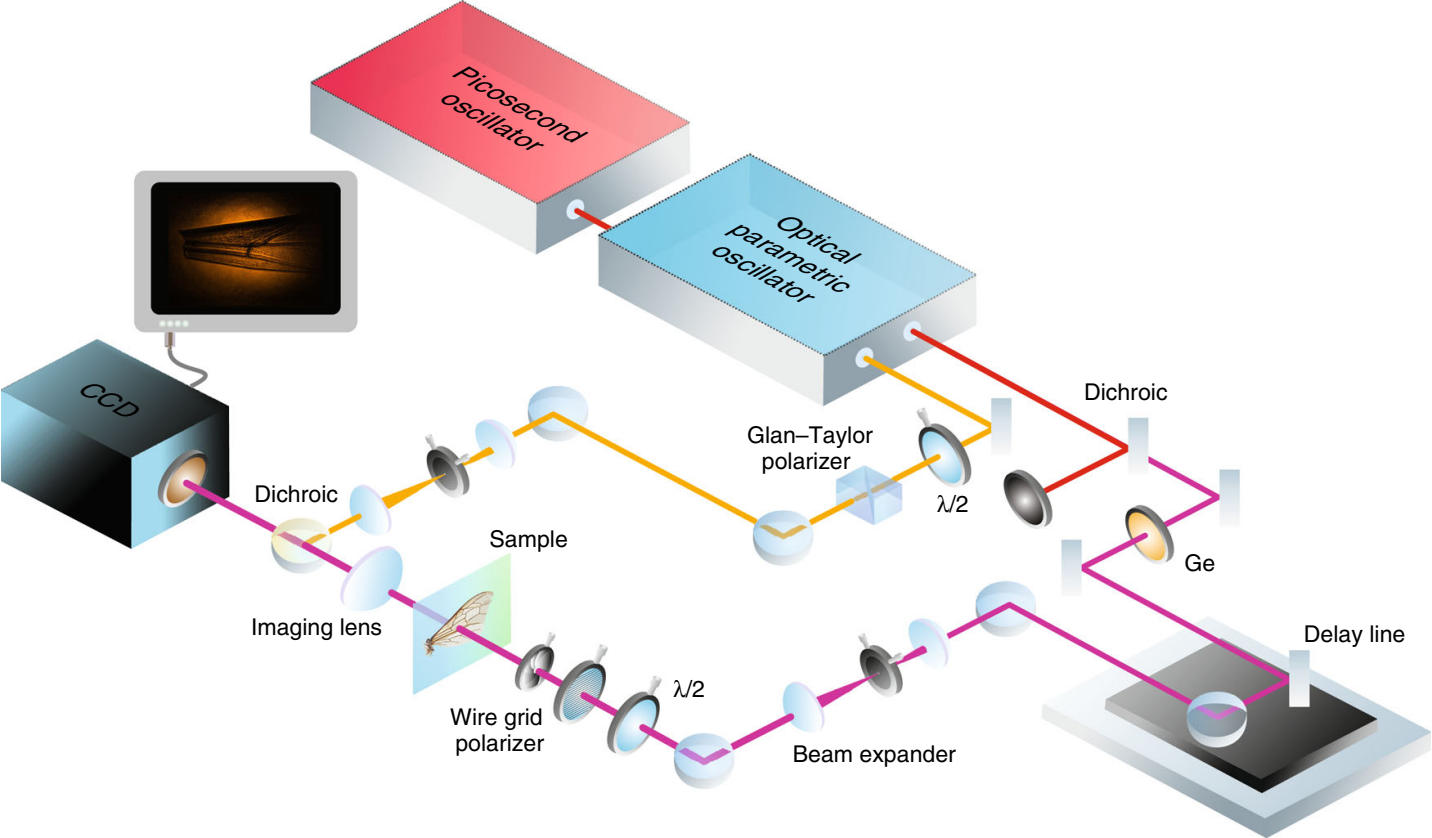
Schematic of a wide-field MIR imaging system based on non-degenerate two-photon absorption in a Si-based CCD camera

Given the excellent NTA performance of a single pixel Si detector, we next explored the feasibility of MIR imaging through direct on-chip NTA in a Si-based CCD camera. Figure 3 provides a schematic representation of the MIR wide-field imaging system based on NTA. The pump and MIR beams are generated by a 4 ps, 76 MHz optical parametric oscillator (OPO) and are expanded to a beam diameter of ~3 mm. The MIR arm contains the sample and a 100 mm CaF_2_ lens to map the image in a 1:1 fashion onto the CCD sensor. The pump beam spatially and temporally overlaps with the MIR beam with the aid of a dichroic mirror so that both beams are coincident on the CCD chip. Note that phase matching is not important for NTA, implying that the angle of incidence of the pump beam can be adjusted freely. Here, we use a conventional, Peltier-cooled CCD camera (Clara, Andor, Northern Ireland) featuring 6.45 × 6.45 μm^2^ pixels in a 1392 × 1040 array. The current magnification and effective numerical aperture of the imaging lens (NA = 0.015) provides an image with ~100 μm resolution, corresponding to ~20 pixels on the camera. Though not the ultimate goal of the current experiments, better spatial resolution can be easily achieved using focusing systems with a higher numerical aperture.

**Fig. 4:**
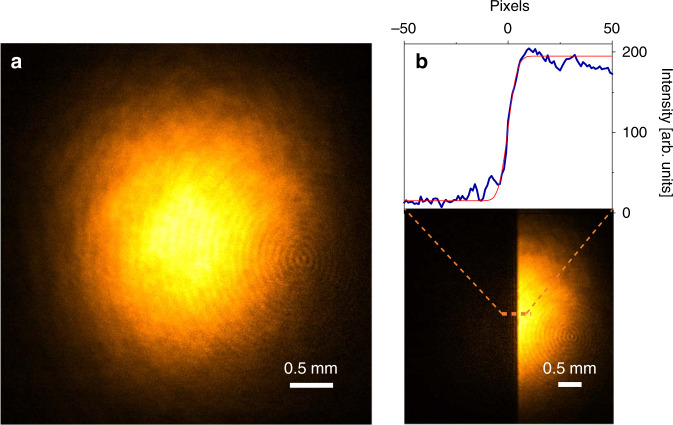
Visualizing the MIR beam profile on a CCD camera. **a** Image of the MIR (3394nm) beam profile using a 1478nm pump pulse. **b** Image of the razor blade covering half of the MIR region. The cross section is shown at the top of the panel. Error function analysis shows that the resolution is ~15 pixels (~100μm) under the current conditions

In Fig. 4a, we show the NTA image of the MIR beam projected onto the CCD sensor using a 1 s exposure time. The degenerate background signal has been subtracted to solely reveal the MIR contribution. With the current experimental arrangement, the background has to be measured only once for a given NIR pump intensity and can be subtracted automatically during imaging, requiring no further postprocessing. See Fig. S2 for a direct comparison of the NIR degenerate background with the NTA signal contribution. Here, we used peak intensities of ~1.5 kW/cm^2^ for the MIR beam and ~1.4 kW/cm^2^ for the NIR pump beam. Under these conditions, each camera pixel only receives pulse energies on the order of a few femtojoules. In Fig. 4b, we show the same MIR beam with a razor blade blocking half of the beam, emphasizing the attained MIR contrast. The fringing at the blade interface is a direct consequence of light diffraction at the step edge. More images of test targets are provided in the Supplementary Information (Fig. S3).

**Fig. 5:**
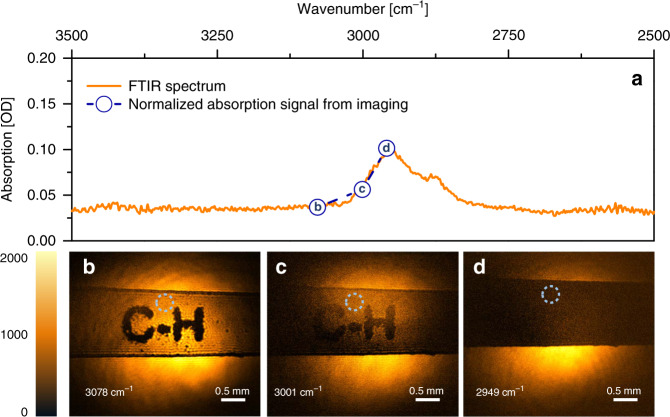
Spectral imaging of a 150-μm thick cellulose acetate film. The printed letters serve as a mask that blocks broadband radiation. **a** FTIR transmission spectrum. MIR image taken at **b** an off-resonance energy, **c** the high energy side of the absorption maximum and **d** the absorption maximum. The exposure time for all images is 1 s

For the current experimental conditions, we find that the MIR intensity changes on the order of 10^−2^ OD in the image are easily discernible even with exposure times shorter than 1 s. To demonstrate the chemical imaging capabilities, we perform MIR imaging on an ~150-μm thick cellulose acetate sheet commonly used as transparencies for laser jet printing. Figure 5a depicts the FTIR spectrum of cellulose acetate in the 2500–3500 cm^−1^ range, showing a clear spectral feature due to C–H stretch vibrational modes. In Fig. 5b, a strip of the cellulose acetate sheet is imaged at 3078 cm^−1^, off-resonant with the C–H stretching vibration. Transmission through the sheet is high because of the lack of absorption. To highlight the contrast, the letters “C–H” have been printed with black ink directly onto the material, providing a mask with limited transmission throughout the 2500–3500 cm^−1^ range. When tuning into the CH-mode resonance (Fig. 5b, 3001 cm^−1^), the transmission is seen to decrease, resulting in lower contrast between the ink and the film. When the MIR is tuned to the maximum of the absorption line (Fig. 5, 2949 cm^−1^), the limited transmission through the film completely eliminates the ink/film contrast. The relative magnitude of MIR absorption, extracted from the images, maps directly onto the absorption spectrum in Fig. 5a, demonstrating quantitative imaging capabilities. The observed contrast is based on a rather modest absorption difference of only 7 × 10^−2^ OD. More examples of MIR imaging of printed cellulose acetate samples can be found in the Supplementary information (Fig. S4).

**Fig. 6:**
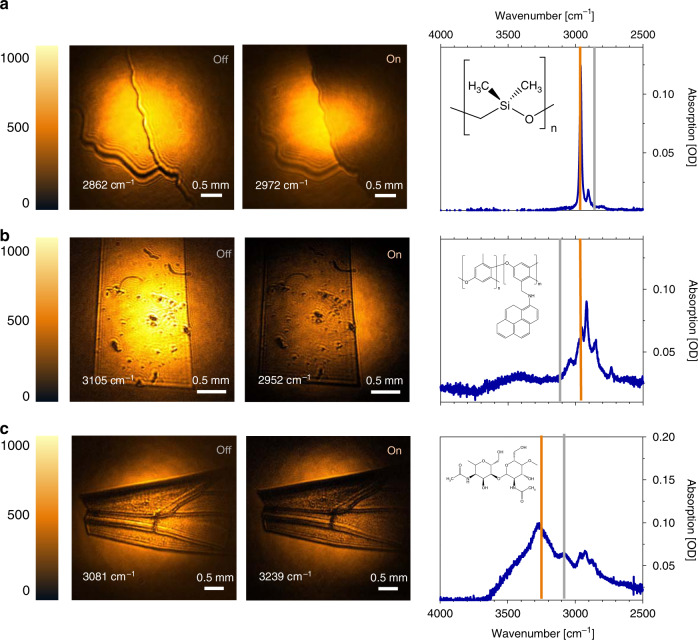
MIR images of various materials accompanied with corresponding FTIR spectra. The left column shows resonance MIR images, whereas the middle column shows MIR images taken at an energy that corresponds with a designated absorptive line. The right column displays the FTIR absorption spectra of the sample with on (orange) and off (grey) resonance frequencies indicated. **a** Interface between D_2_O and silicone lubricant. **b** APPPO polymer film. **c** Wing of a common bee. The exposure time for all images is 1 s

With the wide-field MIR imaging capabilities thus established, we highlight several examples of chemical imaging of various materials. To suppress contrast due to the refractive index differences, we suspended the materials in (vibrationally non-resonant) D_2_O to reveal the true absorption contrast. Figure 6a depicts the interface between D_2_O and an ~20-μm thick polydimethylsiloxane film, a silicon-based organic polymer commonly used as vacuum grease. The difference between the images taken on and off-resonance with the methyl stretching mode reveals clear chemical contrast. Note that the boundary between the polydimethylsiloxane film and D_2_O is evident due to light scattering at the interface. Similarly, in Fig. 6b, chemical contrast is evident when tuning on and off the C–H stretching resonance of a 30 μm membrane of poly(2,6-dimethylphenylene oxide-co-*N*-(2,6-dimethylphenylene oxide) aminopyrene), a material of considerable interest as an ion-exchange membrane. Last, we demonstrate MIR imaging of a bee’s wing in Fig. 6c, a rather complex natural structure that is rich in chitin. The chitin MIR spectrum in the 2500–3500 cm^−1^ range contains overlapping contributions from OH-, NH- and CH-groups, resulting in broad spectral features. The absorption difference between the 3239 and 3081 cm^−1^ vibrational energies is ΔOD = 0.04, yet the contrast difference is still evident from the NTA MIR image.

### MIR videography of the sample dynamic*s*

The signal strength is sufficient for MIR imaging at even faster acquisition rates. In the Supplemental Information, we provide MIR imaging through NTA with a 100 ms exposure time along with an analysis of the pixel noise (Figs. S5–S7). Given that the current camera requires an additional 100 ms of readout time per frame, the effective imaging acquisition time was pushed to 5 fps. Under these conditions, we recorded videos of several mechanical and physical processes as well as live microorganisms. First, in Video 1 (please see Supplementary information), the real-time movement of a printed target on cellulose acetate films is demonstrated, both under vibrational off-resonance (V1a) and resonance (V1b) conditions. Video V2 shows a live recording of the dynamics of a immersion oil droplet placed atop the CaF_2_ window under vibrationally resonant conditions. The flowing droplet can be seen with clear chemical contrast in real time. Moreover, one can observe the formation of intensity fringes near the edge of the droplet due to Fresnel diffraction and interference within the oil film, i.e., Newton’s ring effect.

In Video V3, we show NTA-based MIR imaging of live nematodes suspended in a D_2_O buffer, recorded at 3381 nm (2958 cm^−1^). The image contrast is due to absorption by the methyl stretching vibrations of protein, in addition to refractive effects. The video demonstrates that active, live nematodes can be captured in real time under the MIR illumination conditions used in NTA detection.

## Discussion

In this work, we have shown that the principle of NTA can be extended to MIR imaging by direct on-chip two-photon absorption in a CCD camera. This principle enabled us to acquire images at 100 ms exposure times at femtojoule-level picosecond pulse energies per pixel, experimental conditions that allow wide-field MIR imaging of live, freely suspended organisms at reasonably high frame rates. The use of a CCD camera serves as an attractive alternative to standard MIR cameras, such as cryogenically and electronically cooled MCT detectors. NTA enables good quality MIR images without cryogenic cooling, significantly reducing the complexity and cost of the detector. In addition, NTA-enabled imaging benefits from the mature technology of Si-based cameras, offering robust and affordable detection solutions. These advantages are not at the expense of sensitivity, as previous work based on MIR femtosecond pulses has shown that NTA offers comparable sensitivity to (single pixel) MCT detectors^31^. The NTA process can be used to detect MIR radiation over a very broad range. Other than the steepness of the semiconductor’s band edge, there are no fundamental limitations on the detection spectral range^31,36,43,46^. In fact, higher efficiencies of the NTA process have been demonstrated when tuning towards higher MIR wavelengths near 10 μm, without the necessity of re-alignment, a sensor change or additional data processing.

Unlike other recent methods for improving MIR detection with visible/NIR detectors, our method takes MIR light as its direct input. Photothermal imaging, for instance, relies on MIR-induced optical changes in the sample (expansion, refractive changes), which are subsequently probed with a visible/NIR beam. NTA-based imaging does not rely on secondary effects in the sample due to MIR illumination, as it registers intensity changes in the MIR directly. Similarly, our new NTA imaging approach differs fundamentally from SFG up-conversion techniques. While the latter also uses a visible/NIR camera to generate MIR-based images, the SFG up-conversion mechanism is based on a separate step that converts the MIR light with the help of a pump beam into visible radiation in an external nonlinear optical crystal^29^. To capture a full image, rapid sampling of crystal orientations must be applied to fulfil phase matching, followed by an image reconstruction step. The NTA method approaches comparable imaging performance while using MIR and pump intensities that are an order of magnitude lower. NTA avoids the external light conversion step and thus significantly simplifies the overall imaging system. Because NTA does not rely on phase matching, it can generate images in a single shot and forgoes the need for post-acquisition image reconstruction.

We note that although NTA is related to degenerate two-photon absorption (DTA), none of the results reported here could be achieved through DTA. DTA is a special case of NTA, in which both incident photons have the same energy. Unlike DTA, which has been used for characterizing the temporal widths of NIR pulses through autocorrelation measurements in semiconductor detectors^48,49^, only NTA makes it possible to turn wide bandgap semiconductors into MIR detectors.

The principle of MIR detection with a Si camera through NTA addresses a pertinent issue in MIR imaging, in particular as applied to microscopy. Fast MCT cameras used for this purpose feature a small number of pixels, with 128 × 128 displays being typical, which limits the mapping of large areas and imaging at high definition. Modern Si cameras benefit from much higher pixel numbers while also gaining from optimized readout electronics. Through NTA, these advantages can be ported into the field of MIR imaging, making fast imaging of larger areas possible.

Although the current work shows the feasibility of NTA-based MIR imaging, the approach can be further improved to achieve even better performance. For instance, the 1.5-mm thick silica window in front of our CCD sensor, which shows significant MIR attenuation, can be easily replaced with a window of higher transmittance in this range. Moreover, modern back-illuminated scientific CMOS cameras offer higher quantum efficiencies, higher pixel numbers and faster frame rates, underlining that NTA can be conducted at much higher efficiencies than what is presented here.

In addition, higher NTA efficiencies can be obtained with shorter pulses. The use of high-repetition rate femtosecond pulses would allow imaging at much lower average power while maintaining high efficiency. Detector arrays based on materials other than Si are also interesting for NTA applications. GaAs, for instance, exhibits significantly higher two-photon absorption efficiencies and a steeper band edge absorption than Si, which are both favourable for MIR detection through NTA. Finally, the practical implementation of the NTA imaging technique requires the availability of a pump beam in addition to an MIR source. Although OPO systems constitute a natural choice because of their broadly tunable synchronized pump/idler pulse pairs, recent developments in MIR light source technology promise alternative solutions that are more compact and affordable, including efficient frequency conversion with long wavelength fibre lasers. Such developments will likely improve the practical implementation of NTA-based imaging for a wide range of applications.

## Materials and methods

### FTIR experiments

Conventional infrared absorption spectra were measured using a Jasco 4700 FTIR spectrometer both in transmission and attenuated total reflection (ATR) geometries. For the ATR experiments, the Jasco ATR-Pro One accessory equipped with a diamond crystal was used. The spectra were averaged over 20 scans and were acquired with a 2 cm^−1^ resolution, close to the resolution of the corresponding picosecond NTA experiments.

### Sample handling

Most of the prepared samples were suspended in D_2_O to suppress refractive effects and thus revealed pure absorption contrast. DMSO and silicone lubricant (Dow Corning) were obtained from Sigma-Aldrich and were used without further purification. The sample materials, including the APPPO polymer film and clipped bee wings, were immersed in D_2_O and confined between hermetically sealed 1-mm thick CaF_2_ windows (diameter = 1″). Experiments on cellulose acetate films were performed in air without the use of CaF_2_ windows. *C. elegans* were obtained from Carolina Biological. Nematodes were picked up from agar plates with filter paper, immersed in a phosphate buffered saline D_2_O solution and placed between two CaF_2_ windows spaced by a 50-μm Teflon spacer.

### Non-degenerate two-photon absorption detection with a Si photodiode

We used a conventional Si photodiode (FDS100, Thorlabs) with the parameters described in the Supplementary Information. The transparent window in front of the Si material was removed to improve MIR transmission. The experiments were performed in a pump-probe geometry with the setup depicted in Fig. 3, without utilizing a separate imaging lens in the MIR arm. Both MIR and NIR beams were focused onto the Si photodiode by a f = 100 mm broadband achromat (Trestle Optics)^50^. The NIR intensity was varied by the combination of a half-wavelength plate and Glan-Thompson polarizer. The MIR intensities were controlled by another half waveplate and a wire-grid polarizer. The polarization of both NIR and MIR optical pulses were linear and parallel and were kept constant throughout the experiments. The MIR beam was modulated at 160 Hz by a mechanical chopper, and the NTA signal contribution was isolated by a lock-in amplifier (SR510, Stanford Instruments).

### Imaging using a CCD camera

A Si-based CCD camera (DR-328G-CO2-SIL Clara, Andor) was used. The setup is explained in Fig. 3 of the main text. We used a 1:1 imaging system with an f = 100 mm CaF_2_ lens to project the image onto the CCD camera (Clara, Andor). For live nematode imaging, the imaging system was changed to ×2 magnification using a 4f imaging system composed of an f = 50 mm CaF_2_ lens and an f = 100 mm broadband achromat (Trestle Optics)^50^.

## Supplementary information

Video 1

Video 2

Video 3

Video 4

Video 5

Supplementary information
