# Supplementary material for: Infrared chemical imaging through non-degenerate two-photon absorption in silicon-based cameras
Source: Light Sci Appl. 2020 Jul 20;9:125. doi: 10.1038/s41377-020-00369-6 (PMC7371741; doi:10.1038/s41377-020-00369-6)
Supplement: Supplementary file 6 — Supplementary information [file 41377_2020_369_MOESM6_ESM.docx]

Supplementary information for infrared chemical imaging through nondegenerate two-photon absorption in silicon-based cameras

David Knez^1^, Adam M. Hanninen^1^, Richard C. Prince^2^, Eric O. Potma^1, 2^, Dmitry A. Fishman^1^

*^1^Department of Chemistry, University of California, Irvine, CA 92697, USA*

*^2^Department of Biomedical Engineering, University of California, Irvine, CA 92697, USA*

Supplementary Information

**Table 1. Specifications for Si photodiode experiments**

| MIR pulse width | 4.1 ps |
| --- | --- |
| MIR spot size | 250 μm |
| NIR spot size | 300 μm |
| Detector impedance | 1 MOhm |
| Detector chip size | 5 mm |
| Reverse bias voltage | 12 V |
| MIR wavelength | 3388 nm |
| NIR wavelength | 1480 nm |
| Estimated quantum efficiency for MIR only* | 7*10^-7^ |

*Note that the standard definition of quantum efficiency is not applicable because NTA detection relies on both the number of NIR and MIR photons

**Table 2. Specifications for Si CCD camera experiments**

| MIR pulse width | 4.1 ps |
| --- | --- |
| MIR spot size | 3 mm |
| NIR spot size | 3.5 mm |
| Pixel size | 6.5 μm x 6.5 μm |
| CCD active area | 1392x1040 pixels |
| CCD protective window | Fused silica 1.5 mm |
| Estimated quantum efficiency for MIR only* | 1*10^-9^ |

* Note that the standard definition of quantum efficiency is not applicable because NTA detection relies on both the number of NIR and MIR photons

*Supplemental Figures*

**Figure S1**. Cross-correlation of MIR (3394 nm) and NIR (1478 nm) pulses.


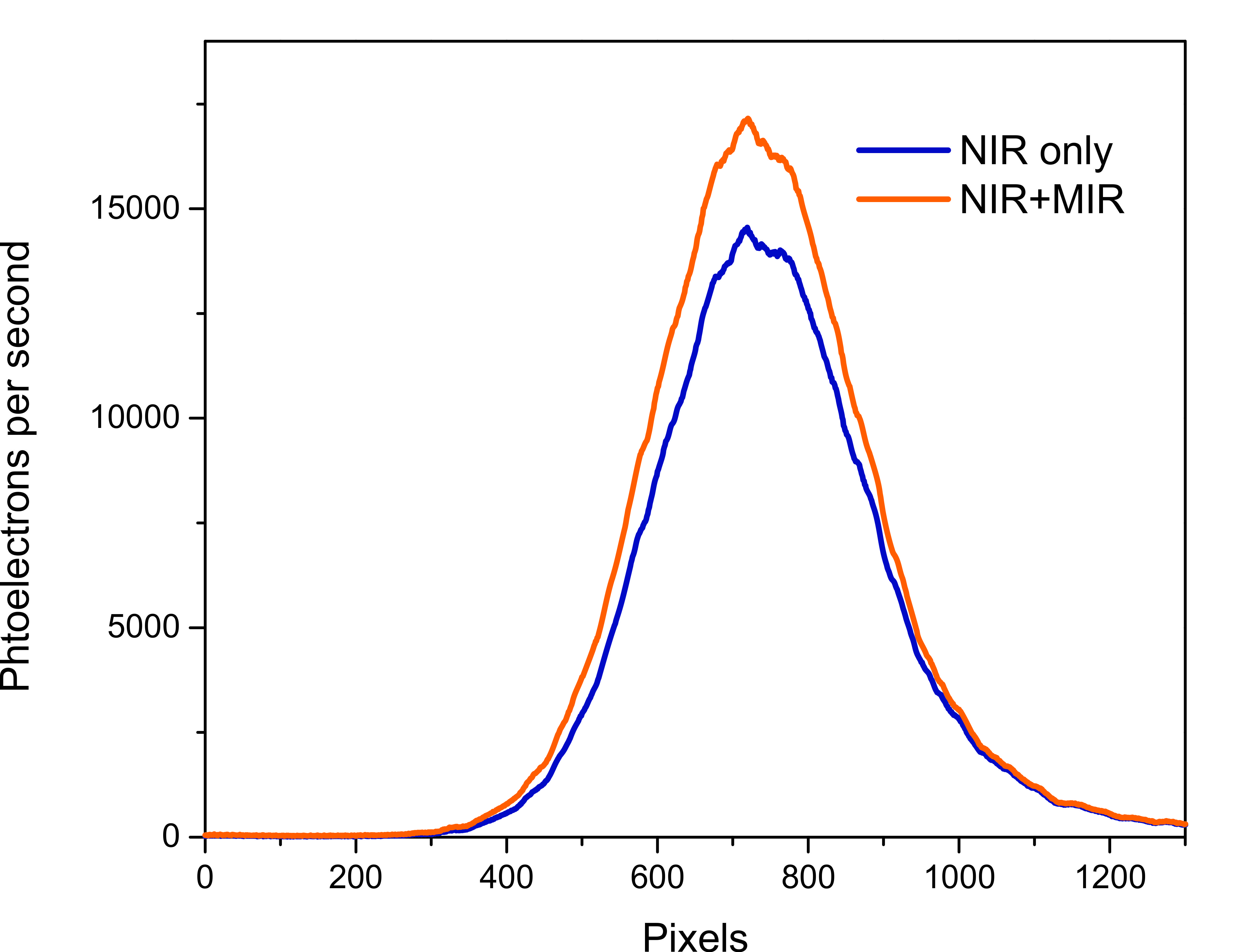


Figure S2. Cross section of beam image: NIR gate pulse only (blue line) and NTA signal (orange line). The ratio of background to signal is ~5 for the given NIR and MIR beam irradiances. The data was retrieved from Figure 4a.


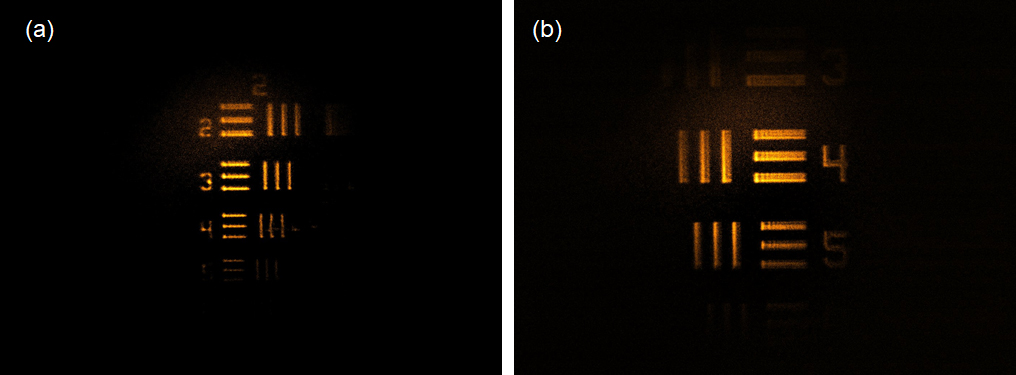


**Figure S3.** Negative USAF 1951 test chart. (a) column 2, (b) column 1. Image was taken at 2947 cm^-1^.


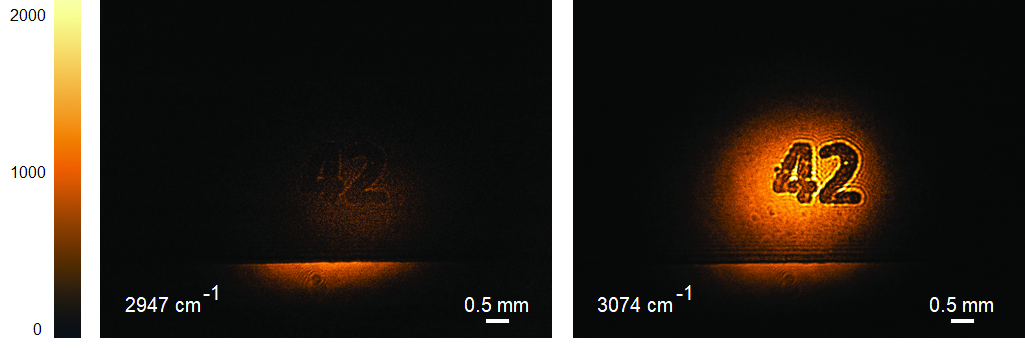


**Figure S4**. Spectral MIR imaging of a 150 μm cellulose acetate film. The numbers printed with black ink serve as mask to indicate contrast.


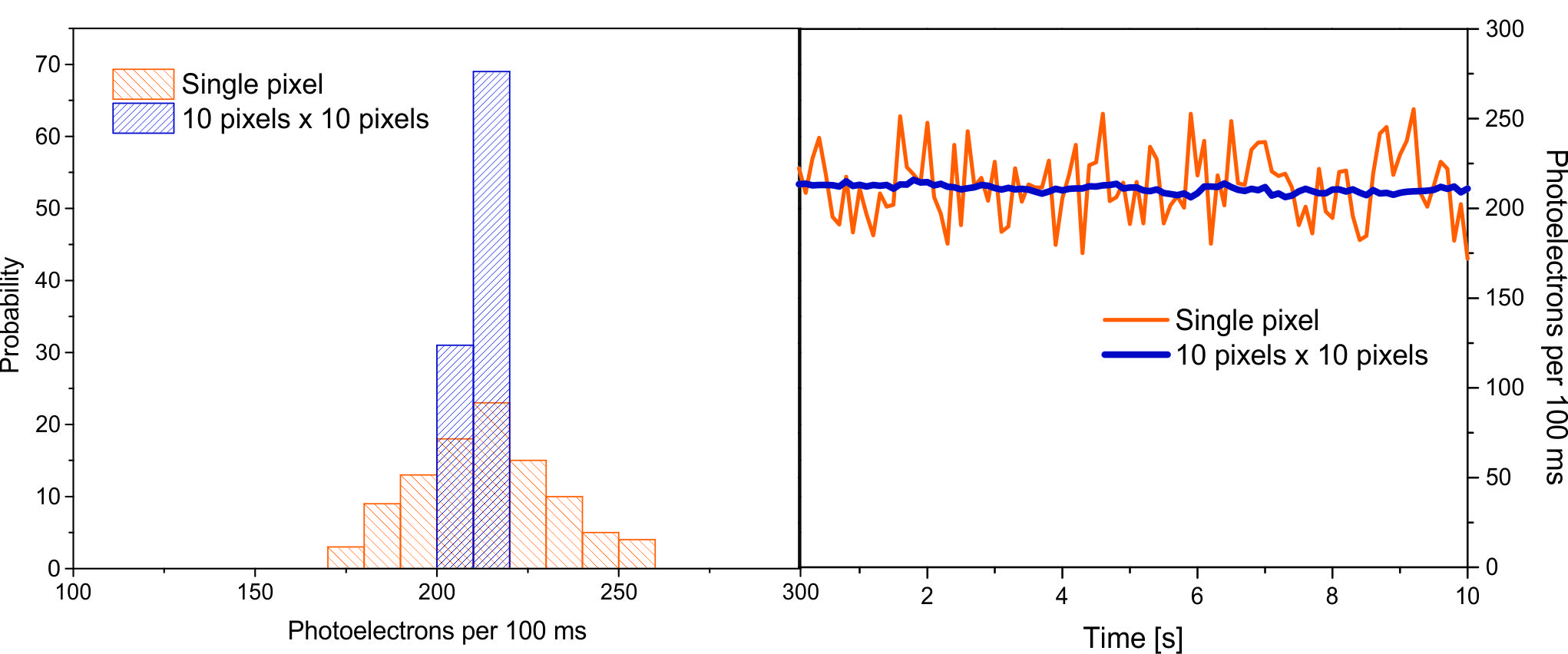


**Figure S5.** Temporal pixel noise in the MIR videos at 100 ms exposure time. For single pixel detection (6.5 x 6.5 μm^2^) of and 2 fJ input energy (without taking into account window attenuation) RMS = 9.1%. For area of 10 pixels x 10 pixels RMS~0.9%. Note that the 10 pixels x 10 pixels area is smaller than spatial resolution of the current imaging system (~15 x 15 pixels ).


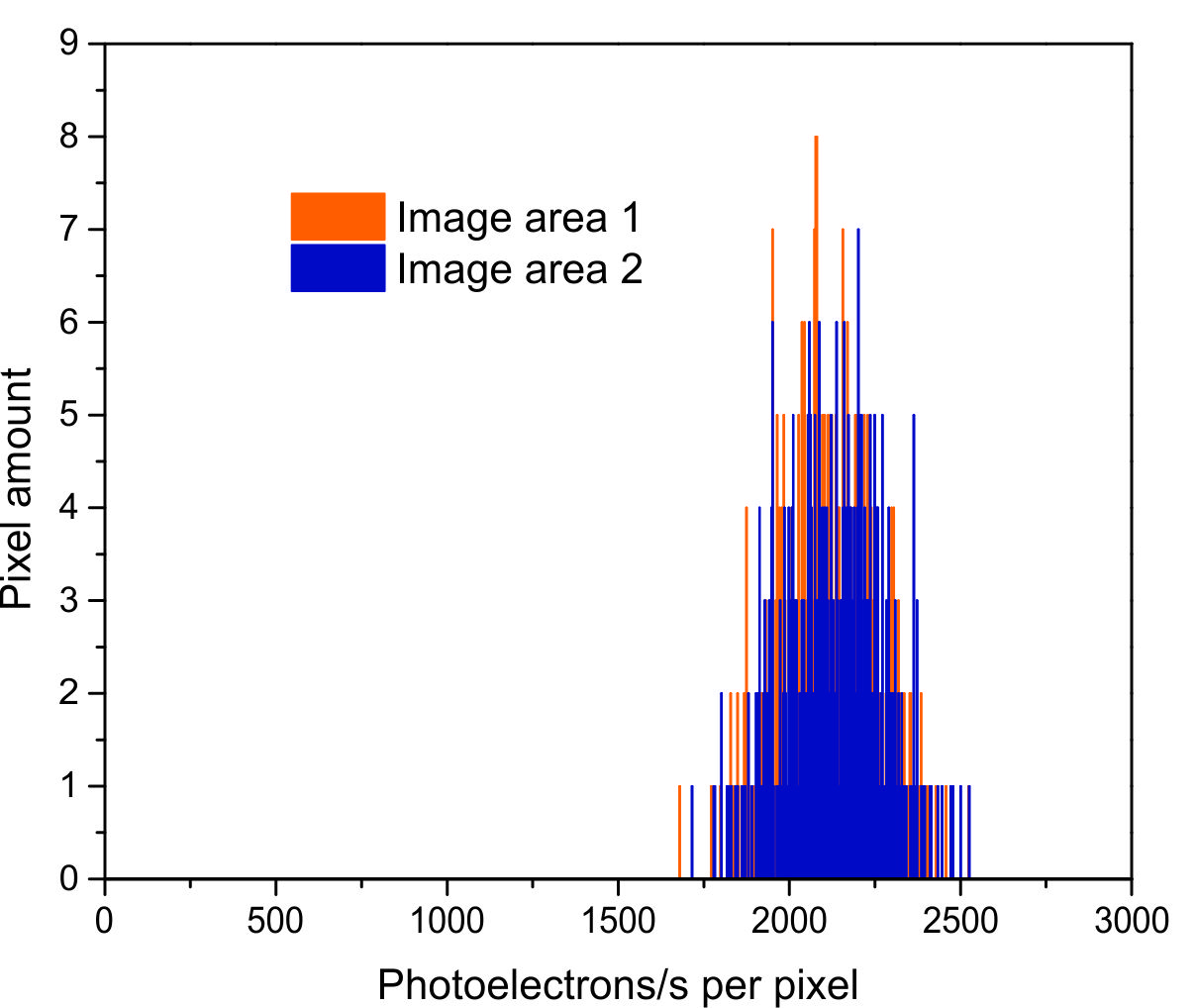


Figure S6. Pixel-to-pixel noise analysis for static imaging with 1 s exposure time. Variance in pixel counts are determined over a region of interest of ~1000 pixels at the center of the beam profile, as presented in Figure 4 of main manuscript. Standard deviation is ~6.4% (Mean: 2100 counts*s^-1^, SD: 135 counts*s^-1^).


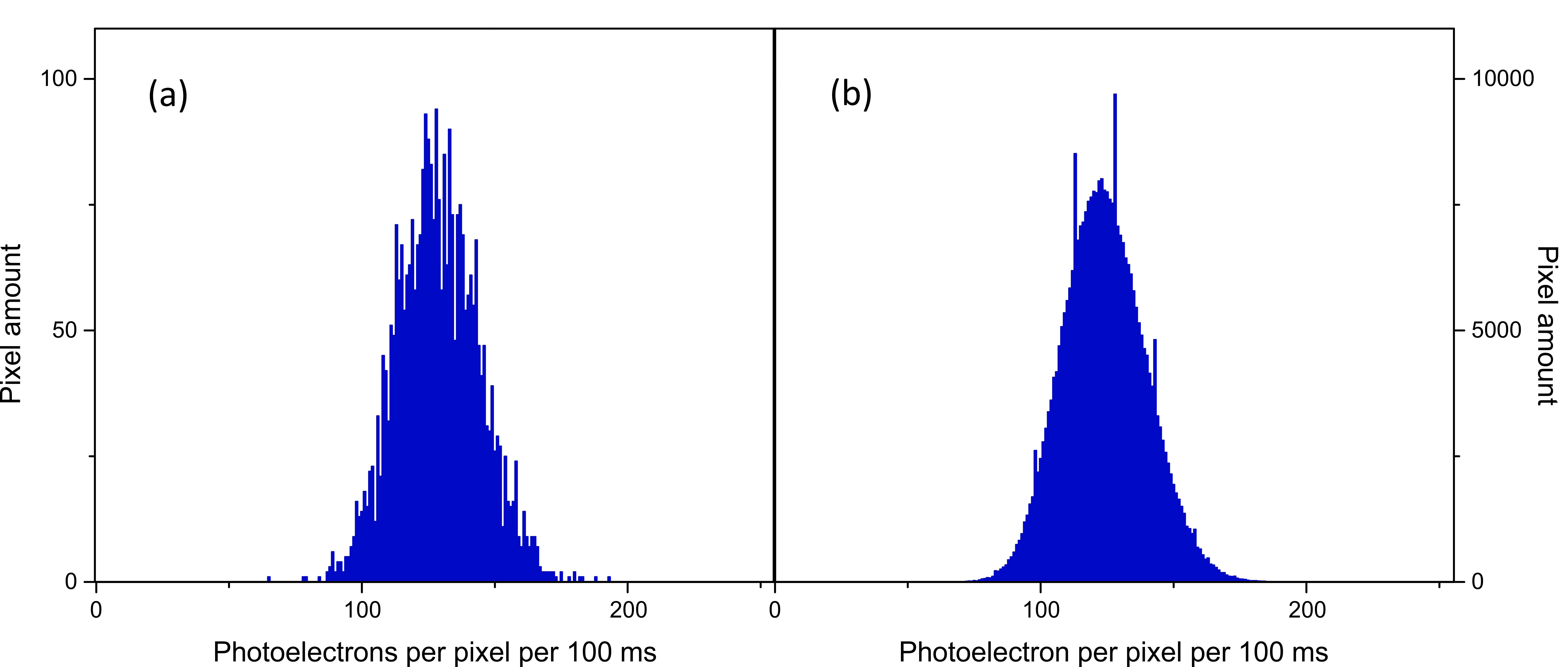


Figure S7. Pixel-to-pixel noise analysis for sequential frames during real time imaging with 100 ms exposure time per frame. Analysis is performed within a region of interest of ~1000 pixels. (a) Single frame statistics at 100 ms exposure time, (b) 100 frame statistics at 100 ms per frame. Both analyses result in ~15% standard deviation.

*Supplemental Movie Files*

**Movie V1**

Real-time movement of a cellulose acetate film strip with printed symbols, both off resonance at 3078 cm^-1^ (V1a) and on resonance at 2949 cm^-1^ (V1b). Movie was acquired with 100 ms exposure time at 5 fps.

**Movie V2**

Real-time movement of an immersion oil droplet on a CaF_2_ surface, on resonance at 2950 cm^-1^. Contrast arises because of MIR absorption by the droplet. Fresnel and Newton-type interference can be seen near the droplet’s edge. Movie was acquired with 100 ms exposure time at 5 fps.

**Movie V3**

Real-time movement of live *C. elegans* nematodes in D_2_O buffer, on resonance at 2950 cm^-1^.

**Movie V4**

Raw real-time movement of live *C. elegans* nematodes in D_2_O buffer, on resonance at 2950 cm^-1^ without subtraction of NIR degenerate pulse beam image. The image is still clearly observed with detailed structure identified. Please, note, that contrast here comes mainly from nematode thickness, which does not exceed few tens of micrometers.
